# Patients Aged 80 or More With Distal Radius Fractures Have a Lower One-Year Mortality Rate Than Age- and Gender-Matched Controls: A Register-Based Study

**DOI:** 10.1177/21514593241252583

**Published:** 2024-05-05

**Authors:** Linnea Arvidsson, Marcus Landgren, Anna Kajsa Harding, Antonio Abramo, Magnus Tägil

**Affiliations:** 1 Department of Clinical Sciences Lund, Orthopaedics, Skåne University Hospital and Lund University, Lund, Sweden; 2 Department of Orthopaedic Surgery, Hand Surgery Unit, 53147 Copenhagen University Hospital, Herlev and Gentofte, Gentofte, Denmark; 3 Department of Hand Surgery, 59568 Skåne University Hospital, Malmö, Sweden

**Keywords:** distal radius fracture, fragility fractures, geriatric trauma, mortality, trauma surgery, upper extremity surgery

## Abstract

**Introduction:**

With a rapidly ageing population, the number of distal radius fractures (DRFs) in the elderly will increase dramatically. The aim of this retrospective register study was to examine the 1- and 5-year mortality in DRF patients aged 80 years or more and correlate the overall survival to factors not related to the fracture itself.

**Material and Methods:**

Patients aged ≥80 diagnosed with DRFs in Lund University Hospital in Sweden in the period 2010-2012 were extracted from the prospective Lund Distal Radius Fracture register. One- and 5-year standardised mortality rates (SMRs) were calculated using the Swedish standard population as a reference. Medical records were searched for non-fracture-related factors including comorbidity, medications, cognitive impairment and type of living. Cox proportional hazard regression models were used to identify prognostic factors for all-cause mortality.

**Results:**

The study cohort included 240 patients, with a mean age of 86. The overall 1-year mortality was 5% (n = 11/240) and the 5-year mortality was 44% (n = 105/240). The 1-year SMR was .44 (CI .18-.69, *P* < .01) when indirectly adjusted for age and gender and compared to the Swedish standard population. The 5-year SMR was .96 (CI .78-1.14). The patients’ ability to live independently in their own home had the highest impact on survival.

**Discussion:**

The 1-year mortality rate among the super-elderly DRF patients was only 44% of that expected. Possibly, a DRF at this age could be a sign of a healthier and more active patient.

**Conclusions:**

The DRF patients aged 80 or more had a substantially lower mortality rate 1 year after fracture compared to the age- and gender-matched standard population. Patients living independently in their own homes had the longest life expectancy. Treatment should not be limited solely because of old age, but individualised according to the patient’s ability and activity level.

## Introduction

The distal radius fracture (DRF) is the most common fracture of the upper extremities.^1^ Advanced age is one of the most important risk factors for DRF and the elderly constitute the largest group of patients with DRFs. In the published studies about DRFs in the elderly, a wide age span is typically presented. Usually, in published series and guidelines, the threshold for being elderly is set to somewhere between 50 and 70 years.^2^ However, defining the elderly as a group this way is imprecise and differences do exist between a 60 and an 80-year-old patient regarding both expectations and needs as determinants of successful outcome.

The number of patients aged 80 years or more is calculated to rise by 50% in Sweden between 2023 and 2030.^3^ With an increasing life expectancy, an exponential increase of osteoporosis with age^4^ and the fact that more people live very active lives even in old age,^5-7^ the number of DRFs in the elderly can be expected to rise. Therefore, we face a major challenge regarding how to optimize both the use of resources and the outcome for our oldest patients with a DRF.

The mortality rates are found to be increased after hip fractures. In a register study of 55 716 patients, the 1-year mortality after a hip fracture was estimated to be 26%.^8^ In a study of patients aged 65 years or older, a fragility fracture occurring at any site, was associated with increased mortality up to 6 years post-fracture. The mortality rate was mainly affected by a hip or a vertebral fracture.^9^ In DRF patients, the results are less clear and the mortality has been shown to be both increased,^10,11^ decreased,^12,13^ or without difference.^14-16^

Previously, the mortality rate in Sweden after a DRF was calculated in 23 394 patients aged 18 years or more in the Swedish fracture register, and the overall 1-year mortality was 2.9%.^17^ However, this study includes patients from all adult age categories. To our knowledge the survival rates after a DRF in patients aged over 80 in Sweden is unknown and is speculatively related to factors other than the fracture.

### Aim of this Study

Our aim was to calculate the one- and 5-year mortality rate for DRF-patients aged 80 years or older, by using a large local DRF register and to correlating the overall survival to non-fracture-related factors.

## Material and Methods

### The Lund Distal Radius Fracture Register

The Department of Orthopaedics, at Skåne University Hospital in Lund, Sweden, serves a population of 300 000 inhabitants from both urban and rural areas. In the prospective Lund Distal Radius Fracture Register, all patients aged 18 years or older with a DRF have been prospectively registered and followed since 2002.^18,19^ Weekly, a secretary scrutinises the medical records from the emergency department for wrist fractures coded as s52.50 and s52.60 according to the International Classification of Diseases 10th Revision (ICD-10). An orthopaedic surgeon examines the X-rays of all included patients to make sure the diagnosis and coding are correct, and then they are included in the register.

### Options for Treatment

Non-displaced fractures are treated in a forearm cast for 4 to 5 weeks. Displaced fractures are reduced and casted. If impossible to reduce primarily, or if secondary loss of reduction occurred at the 7-10 day follow-up, surgical treatment is recommended. However, the final decision lies with the responsible surgeon and the patient. In most surgically treated, a volar locking plate (VLP) is used for osteosynthesis. The treatment protocol has remained the same for all ages during the whole period.^18^

### Patients

In the present study we extracted a subset of patients from the Lund distal fracture register. All patients in the register who were at least 80 years old at the time of injury and had their DRF between January 1, 2010 and December 31, 2012 were included. We had no exclusion criteria. Patients were followed from the incidence date (time of fracture) to the date of death or the closing date of the study, which was set to January 1, 2020.

The inclusion years were set to 2010-2012 to enable a long follow up for all patients. The closing date was set to before the outbreak of the COVID-19 pandemic to minimize the risk of our results being affected by the potential excess mortality during this period.

Data regarding age, gender, date of injury, treatment date and type of surgery was extracted from the register. The medical records were reviewed for comorbidity, use of medication, cognitive impairment, type of living and use of walking aids. If the information was missing in the medical records at the date of injury, notes up to 1 year back in time were read in order to retrieve the data. To see if the patients suffered from major complications, all medical records were reviewed to discover whether the patient had visited the orthopaedic, hand surgery or radiology unit again, due to complaints from the same wrist that had suffered the DRF. We did not look for minor complications.

### The Non-fracture Related Factors

The Charlson Comorbidity Index (CCI) was used to classify comorbidity. In this index a number of common chronic conditions are scored with weighed values of 1, 2, 3 or 6, depending on severity and influence on mortality. A weighed value of 1 represents a slightly elevated mortality rate and 6 a very high mortality rate.^20^ Due to the relatively limited cohort, the patients in our study were dichotomised into Charlson index “less than 2” or “2 or more”. In the original CCI, age is also taken into account when calculating points; in this study we have only given points for comorbidities. Cognitive impairment was also analysed as a separate variable (apart from “dementia” as one of the categories in the Charlson index) and included both patients with a dementia diagnosis as well as patients reported in the medical records as having a cognitive impairment without having a neurodegenerative diagnosis. The number of prescribed drugs was dichotomised into “5 or less” or “polypharmacy” (more than 5). Type of living was categorised as “Independent living”, “Living with assistance” or “nursing home” (this also including dementia care facilities). Information on the use of walking aids (cane, crutches, walker) was taken from the medical record and coded as yes or no.

### Statistical Analysis

All statistical analyses were made using IBM SPSS statistics for Mac V.28.0.0. The authors performed the statistical analysis. A two-sided *P*-value <.05 was considered significant. Scale variables for patient characteristics had a symmetrical distribution and are presented as mean with standard deviation. Categorical data is presented with count and proportions (%).

Dates of death were extracted by linking the social security number for each patient to their death certificates in the Swedish National Cause of Death register. The crude 1- and 5-year death rates were calculated by the Kaplan-Meier method. The standardised mortality ratio (SMR) is expressed as a percentage quantifying the decrease or increase in mortality of a study cohort compared to the general population. If lower than 1, there is a lower number of deaths than expected. The SMR also constitutes an indirect form of standardisation. In this study, age and sex standardised SMRs were calculated. The mortality rates of the standard Swedish population for the same period of time was used as a reference population.^21^ Age groups used in the indirect standardisation were 80-84, 85-89, 90-94 and 95-99. Cox proportional hazard regression models were used to identify prognostic factors of all-cause mortality, presented as hazard ratio (HR) with 95% confidence intervals (CI). Analyses were based on time-to-event, where time is the time from the date of fracture and event is defined as all-cause mortality. The primary analysis aimed to determine the risk factors associated with all-cause mortality. The potential prognostic factors in the analysis were the independent variables: age, gender, comorbidities according to the CCI, polypharmacy, cognitive impairment, type of living and use of a walking aid. Multivariate Cox proportional hazard models were used to adjust for interactions between prognostic factors of mortality. Kaplan-Meier estimates with log rank tests were used to plot survival and compare survival between stratified groups.

## Results

### Patients and Treatment

Between January 1, 2010, and December 31, 2012, in total 240/1317 (18.2%) DRF patients in the register were 80 years or older at the time of their fracture. See patient characteristics in Table 1. During the time period, 308/1317 (23.4%) of all the DRF patients in the register (age 18-96) were treated surgically, compared to 25/240 (10.4%) in the subset of patients aged 80 years or more. Among the subset of elderly, the majority of the surgically treated patients underwent open reduction and internal fixation using VLPs (n = 18). Other methods used were fragment-specific fixation (n = 2) and external fixation (n = 3). After completion of treatment, three of the patients sought care again due to pain from the injured wrist. All three had been treated non-surgically, and all three suffered from malunion with more than 30 degrees of dorsal angulation. One of the patients underwent an osteotomy.

**Table 1.** Patient Characteristics.

Characteristics	Patients n = 240 (%)	Missing Values
Age		
Mean	86 (SD 4.2)	
Range	80-97	
Gender		
Males	26 (11)	
Females	214 (89)	
Charlson comorbidity index		
0	56 (23)	1
1	82 (34)	
2-3	86 (36)	
4+	15 (6)	
Polypharmacy		
Yes	127 (53)	26
No	87 (36)	
Cognitive impairment		
Yes	43 (18)	43
No	191 (82)	
Type of living		
Own housing with no assistance	136 (57)	18
Own housing with assistance	45 (19)	
Nursing home	41 (17)	
Walking aid		
Yes	81 (38)	29
No	130 (62)	

Baseline characteristics of the patients in the study cohort.

### Mortality

During the study period, 74% (178/240) of the patients in the cohort died. No difference was found in overall survival between men and women. From the date of fracture, the mean survival time of the patients was 5.2 years (95% CI 4.8-5.6). The crude overall 1-year mortality was 5% (n = 11/240) in the study cohort and 11% (n = 54 309/496 904) in the standard population. The crude 5-year mortality was 44% (105/240) in the study cohort and 55% (n = 271 886/496 904) in the standard population.

The 1-year age and sex standardised SMR for the DRF patients aged 80 or more was .44 (95% CI .18–.69, *P* < .01); thus, in the short term, the mortality rate was only 44% of that expected when compared to the Swedish general population and weighted for both age and gender. The 5-year age and sex standardised SMR was the same as expected in the general population: SMR .96 (95% CI .78–1.14).

In the univariate Cox regression analysis, assisted living or living in a nursery home, a high rate of comorbidity, cognitive impairment and polypharmacy were significantly associated with a poorer prognosis. There were no significant associations between overall survival and gender, increasing age or use of a walking aid.

**Figure 1.**
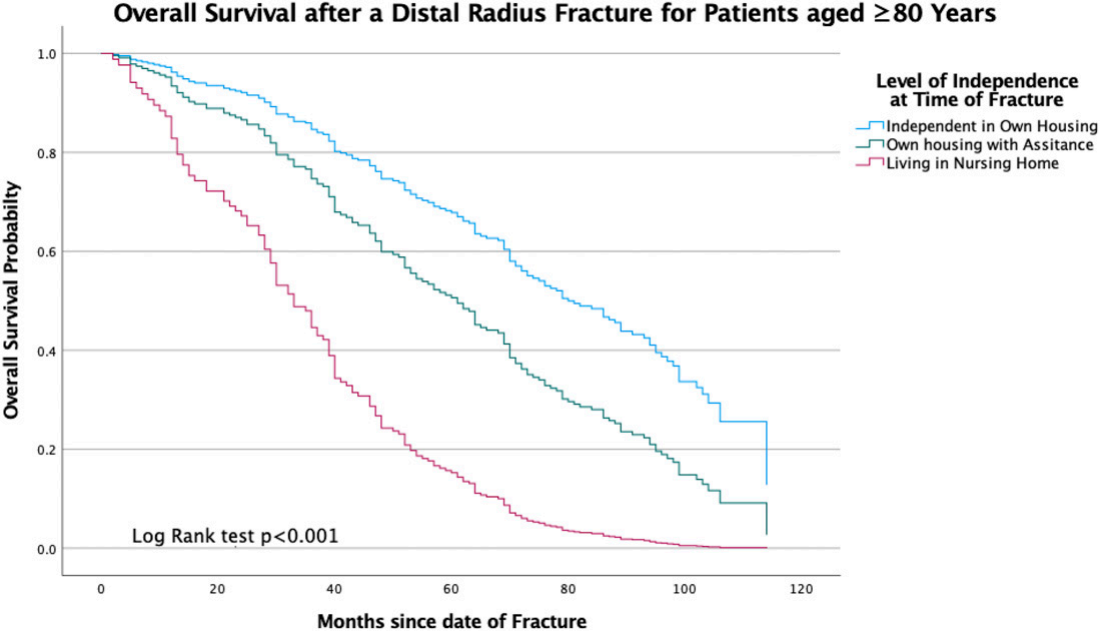
Kaplan-Meier plot for overall survival, for patients aged 80 years or more with distal radius fracture. Time (months) from fracture date. Blue line: patients living in own housing, green line: patients living in own housing with some type of regular assistance, red line: patients living in nursing home or equivalent care facility.

In the adjusted multivariate Cox regression model, the best predictor of mortality was the type of living. Patients with some type of regular assistance at home had a poorer prognosis compared to fully independent patients living in their own home (HR 1.6, 95% CI 1.0–2.4, *P* < .05). Patients living in a nursing home had the poorest prognosis (HR 3.2 95% CI 1.8–5.6, *P* < .001). The mean survival of patients living in their own home was 6.4 years (95% CI 6.0–6.9) compared to patients in nursing homes with a mean survival of 3.1 years (95% CI 2.5–3.7, *P* < .001). The difference in survival expectancy of patients with different living situations is displayed in a Kaplan-Meier estimation; see Figure 1. In the adjusted multivariate Cox regression model, a CCI score of 2 or more also affected the prognosis (HR1.5 95% CI 1.0–2.2, *P* < .05). Cognitive impairment and polypharmacy showed no significant impact on survival in the adjusted model.

## Discussion

Our main finding was, somewhat surprisingly, that patients 80 years or older had a substantially lower mortality one year after a DRF compared to the age- and gender-matched standard population. This finding differs from several previous reports. However, since the majority of previous studies analysed a wider range of age categories it is difficult to accurately compare the results.

In an American cohort of 325 patients aged 65 years or older, the mortality rate was significantly higher after a DRF than in the standard US elderly population. The estimated survival was 57% 7 years after the fracture, i.e., less than the expected 71% for elderly US residents.^10^ In a Norwegian cohort, 883 patients with a DRF were stratified for age, and an increased mortality was seen, but only in female patients aged 70 years or older.^11^ When analysing patients aged more than 80 in subgroups Kwon et al found a subtle decrease in 1-year mortality (SMR .88) after a DRF-fracture among patients over 90 in a Korean cohort. However, the mortality was unaffected for patients 80-89 years.^12^

Chung et al found lower mortality rates in DRF patients when analysing standardised mortality rates in 81 568 patients aged 65 years or older in the US Medicare population. The authors suggested that regular visits to a healthcare provider after a DRF could be a possible explanation, allowing diagnosis and treatment of other conditions contributing to mortality.^13^ In our cohort, 90% of the patients were treated non-surgically with casts and received only 1 or 2 return visits without further investigation regarding medical conditions, so in our series we find it difficult to see this as an explanation.

One can speculate that a DRF in our cohort was simply an indirect sign of a healthier patient. A DRF could be an indicator of a better balance and a functioning reflex system enabling the patient to fall on the outstretched hand instead of landing on for example the hip. A DRF could also indicate a relatively active lifestyle as it is necessary for the patient to be reasonably mobile in order to sustain a fracture.

In our study, the difference between the mortality in the cohort and the reference population decreased over time. Even though the patients aged 80 or more had a great chance of living another year, the chance of living many more years decreases significantly per year in higher age. This could explain the fact that the differences no longer appear in the 5-year analysis, i.e., this may happen because the expected average survival is evened out over time among the oldest in the population.

The best marker of expected survival was type of accommodation which could be seen as an indirect indication of the patient’s level of autonomy. The low mortality in this study was not explained by a lower proportion of patients living in their own homes. In the same geographic area as the hospital serves, statistics from the National Board of Health and Welfare show that 15% of those over 80 lived in nursing homes or residential care facilities in 2014,^22^ while that number was 17% in this study.

With limited resources in healthcare and a large and increasing number of patients, we should consider how we allocate resources and which patients benefit best from surgical treatment. For a long time, the perception has been that the oldest patients do not benefit from advanced care for DRFs, and that their recovery will be “good enough” regardless of the type of treatment, but is this really true? In recent years, opinions seem to have shifted as more and more studies demonstrate that elderly patients benefit from surgical treatment.

In elderly DRF patients, anatomically pre-contoured VLPs are the most commonly used implant for osteosynthesis.^23,24^ Elderly patients treated with VLPs have a better functional outcome than the conservatively treated^25,26^ and a similar early complication rate as in younger patients.^25,27^ It has been shown that surgery can lead to a faster start of range of motion training and an earlier increase in grip strength,^25^ leading to a quicker return to regular activities.^2^ For an old patient, time is a scarce commodity and early activation and return of hand function could allow the patient to maintain independence, probably resulting in a lower overall cost for the health care and society. The high mortality following a hip fracture has created an interest in improving the care for these patients with multidisciplinary care.^28-30^ Perhaps this could also benefit the group of frail elderly with DRFs.

Maybe the treatment of DRFs, like other diagnoses in elderly, should be more individualised, and should reflect functional needs as well as physical capacity to go through an operation. Possibly, we should consider surgical treatment of more elderly to enable those who are active with high demands on their wrist function, to continue to live independent lives. Depending on the functional level, different types of rehabilitation and follow-up may also be relevant^31^ regardless of whether surgical treatment is provided or not. It is obvious, particularly in patients aged 80 or more, that health-related factors such as comorbidity and level of independence are more relevant for assessing future needs for good wrist function, than the number of years previously lived.

## Limitations

A limitation of the study is the relatively small cohort size and the low number of male patients. Even if the standardised mortality rate is adjusted for gender, the tendency is that survival mostly reflects the survival of women since they constitute 89% of the patients. The study has not taken into account possible differences in socio-economics. However, the hospital’s catchment area spans both cities and rural areas, and the facility is the only one in the area that treats acute DRFs. The socio-economics are therefore expected to be varied and at least relatively comparable to the entire country. No power analysis was conducted before the study. To describe the uncertainty of each estimate the CIs are presented with each *P*-value. There are some missing data concerning patient characteristics that could affect the HRs in the Cox regression estimations; this was due to missing information in the medical records. However, our main aim in the study was to calculate the mortality rates of the patients and for this the dataset is complete.

## Conclusions

This study is the first to evaluate both short- and long-term survival among DRF patients aged 80 or more. Our main finding was that these elderly patients had a substantially lower mortality one year after the fracture compared to the age- and gender-matched standard population. The best predictor of mortality was the patient’s type of living, where living in a nursery home had the worst prognosis.

In conclusion, age alone appeared inappropriate for triaging the patients aged 80 or more to best possible treatment. In fact, the patients in this study seemed to have passed a threshold where age no longer affected mortality on an annual basis. The patients ranged from healthy independent patients with high demands and high expectations to dependent patients with dementia, unlikely to cooperate in a strenuous rehabilitation. However, a high percentage of the patients seemed to have many years left to live, where a functional and pain-free wrist could be decisive for the quality of life.

## Data Availability

Data supporting the findings of this study are available from the corresponding author upon reasonable request.*
